# The association between fine particulate matter exposure during pregnancy and preterm birth: a meta-analysis

**DOI:** 10.1186/s12884-015-0738-2

**Published:** 2015-11-18

**Authors:** Xiaoli Sun, Xiping Luo, Chunmei Zhao, Rachel Wai Chung Ng, Chi Eung Danforn Lim, Bo Zhang, Tao Liu

**Affiliations:** 1 Gynecology Department, Guangdong Women and Children Hospital, No. 521, Xingnan Road, Panyu District, Guangzhou, 511442 China; 2 Sydney South West Clinical School, Faculty of Medicine, University of New South Wales, Sydney, Australia; 3 Faculty of Science, University of Technology Sydney, Ultimo, Australia; 4 Department of Preventive Medicine, School of Public Health, Sun Yat-sen University, Guangzhou, 510080 China; 5 Guangdong Provincial Institute of Public Health, Guangdong Provincial Center for Disease Control and Prevention, No. 160, Qunxian Road, Panyu District, Guangzhou, 511430 China; 6 Environment and Health, Guangdong Provincial Key Medical Discipline of Twelfth Five-Year Plan, Guangzhou, 511430 China

**Keywords:** Fine particulate matter, Preterm birth, Meta-analysis, Adverse pregnancy outcome

## Abstract

**Background:**

Although several previous studies have assessed the association of fine particulate matter (PM_2.5_) exposure during pregnancy with preterm birth, the results have been inconsistent and remain controversial. This meta-analysis aims to quantitatively summarize the association between maternal PM_2.5_ exposure and preterm birth and to further explore the sources of heterogeneity in findings on this association.

**Methods:**

We searched for all studies published before December 2014 on the association between PM_2.5_ exposure during pregnancy and preterm birth in the MEDLINE, PUBMED and Embase databases as well as the China Biological Medicine and Wanfang databases. A pooled OR for preterm birth in association with each 10 μg/m^3^ increase in PM_2.5_ exposure was calculated by a random-effects model (for studies with significant heterogeneity) or a fixed-effects model (for studies without significant heterogeneity).

**Results:**

A total of 18 studies were included in this analysis. The pooled OR for PM_2.5_ exposure (per 10 μg/m^3^ increment) during the entire pregnancy on preterm birth was 1.13 (95 % CI = 1.03–1.24) in 13 studies with a significant heterogeneity (Q = 80.51, *p* < 0.001). The pooled ORs of PM_2.5_ exposure in the first, second and third trimester were 1.08 (95 % CI = 0.92–1.26), 1.09 (95 % CI = 0.82–1.44) and 1.08 (95 % CI = 0.99–1.17), respectively. The corresponding meta-estimates of PM_2.5_ effects in studies assessing PM_2.5_ exposure at individual, semi-individual and regional level were 1.11 (95 % CI = 0.89–1.37), 1.14 (95 % CI = 0.97–1.35) and 1.07 (95 % CI = 0.94–1.23). In addition, significant meta-estimates of PM_2.5_ exposures were found in retrospective studies (OR = 1.10, 95 % CI = 1.01–1.21), prospective studies (OR = 1.42, 95 % CI = 1.08–1.85), and studies conducted in the USA (OR = 1.16, 95 % CI = 1.05–1.29).

**Conclusions:**

Maternal PM_2.5_ exposure during pregnancy may increase the risk of preterm birth,but significant heterogeneity was found between studies. Exposure assessment methods, study designs and study settings might be important sources of heterogeneity, and should be taken into account in future meta-analyses.

**Electronic supplementary material:**

The online version of this article (doi:10.1186/s12884-015-0738-2) contains supplementary material, which is available to authorized users.

## Background

Preterm birth (before 37 weeks of gestation) is the leading cause of newborn deaths and the second-leading cause of death (after pneumonia) in children less than 5 years old [1]. More than 1 million children die each year worldwide due to complications of preterm birth. Many survivors face lifelong disabilities and chronic diseases, including learning disabilities, adult hypertension, diabetes, coronary heart disease, and visual and hearing problems [1, 2]. An emerging body of evidence indicates that ambient air pollution may play an important role in the incidence of preterm birth [3, 4]. As a prominent component of the ambient air pollution mixture, fine particulate matter (PM_2.5_, aerodynamic diameter <2.5 μm) may cause greater harm to human health due to its specific characteristics such as smaller diameter, larger surface area, and longer suspension time in air [5, 6]. Although previous studies have estimated the association between PM_2.5_ exposure during pregnancy and preterm birth, the results have been inconsistent and remain controversial [7–10].

To quantitatively summarize the association between PM_2.5_ exposure and preterm birth risk, a few meta-analyses have been conducted during the past several years [11–13]. However, due to some methodological issues in previous studies, further research is needed. For example, all three meta-analyses found a significant heterogeneity between included studies [11–13]. According to the Cochrane guide, it is not appropriate to simply combine the results of articles with significant heterogeneity [14]. Although some authors have recognized this issue in their studies, the limited number of included studies prevented them from quantitatively testing the sources of heterogeneity [11, 12]. In the past several years, more studies have been conducted to estimate the association between maternal PM_2.5_ exposure and preterm birth, which provides an opportunity to quantitatively explore the sources of heterogeneity between previous studies and meta-analyses.

In this study, we collected previously published studies that assessed the association between PM_2.5_ exposure during pregnancy and preterm birth, and employed a meta-analysis model to quantitatively evaluate the effects of PM_2.5_ exposure during different phases of pregnancy on preterm birth. We further explored the modification of exposure measurement methods, study settings and study designs on the meta-estimates of PM_2.5_.

## Methods

The methods for the analysis and inclusion criteria were specified in advance and documented in a protocol.

### Literature search

We searched for all publications indexed in the MEDLINE, PUBMED and EMBASE databases as well as the China Biological Medicine and Wanfang databases during November and December 2014. The search strategies used combinations of the following key words: “air pollution”, “particulate matter”, “fine particulate matter”, “fine particles”, “PM”, “PM_2.5_”, “PM _2.5_”, “premature birth”, “preterm birth”, “PTB”, “preterm delivery”, “PTD” and “prematurity”. We also manually searched the references of every primary study for additional publications. Further publications were also identified from review articles. Only publications in English or Chinese were considered.

### Inclusion and exclusion criteria

**Fig. 1:**
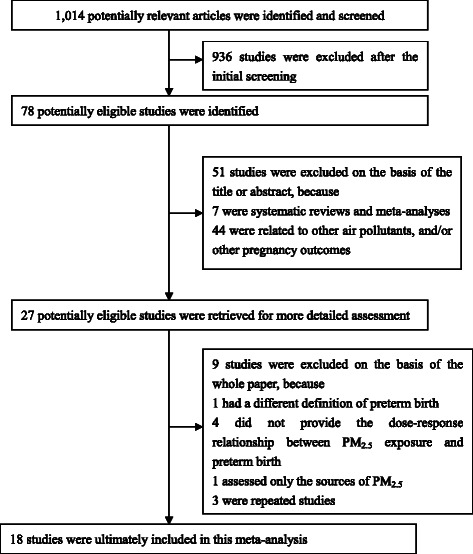
Flow chart of the study selection process

We initially screened the titles and abstracts of all studies. Studies were excluded if they were not related to fine particulate matter and preterm birth. The remaining studies were noted as potentially eligible studies and were further viewed by two independent authors. The studies were included in this meta-analysis if they met the following criteria: (a) studies included PM_2.5_ exposure during pregnancy and preterm birth that was defined as a live birth before gestational week 37; (b) studies presented sample sizes and odds ratios (OR) with 95 % confidence intervals (CI) or information that could be used to infer these results; (c) if more than one study was identified for the same population, only the study that included the most recent population or the most information was selected. Studies that did not meet the above criteria were excluded. The process of study selection is presented in detail in Fig. 1.

### Data extraction

The following information was extracted from each study: authors, year and source of publication, study period, study setting, study design, PM_2.5_ exposure assessment methods, data sources, sample size, PM_2.5_ exposure windows, exposure range, and ORs and 95 % CIs. If a study provided associations between preterm birth and PM_2.5_ exposure during the entire pregnancy and trimester-specific periods, all estimates were extracted. Several studies assessed PM_2.5_ exposure based on monitoring network data and remote sensing data; we preferentially chose estimates based on monitoring network data because this assessment method was more common across all studies, which could potentially reduce the heterogeneity between studies in this meta-analysis. In addition, because there is considerable co-linearity between pollutants originating from the same sources and not all studies adjusted for air pollutants other than PM_2.5_, we extracted estimates only from single pollutant models fully adjusted for other covariates. Eligibility assessment and all data extraction were conducted by two authors using a standard form, and discrepancies were resolved by discussion between authors. The authors adhered to PRISMA guidelines for meta syntheses. Ethical approval was not required for this meta analysis. We employed the New Castle Ottawa scale to assess the quality of all included studies [15]. For the retrospective studies, the quality assessment was based on participant selection, comparability and exposure assessment; For the prospective studies, the quality assessment was based on participant selection, comparability and outcome.

### Meta-analysis and statistical analysis

Prior to performing the meta-analysis, we converted all ORs to a common exposure unit of 10 μg/m^3^ increase in PM_2.5_ exposure, which allowed us to quantitatively pool estimates from different studies. Firstly, all ORs and their 95%CIs were converted by logarithms (ln), which were used to calculate the partial regression coefficients (β) and their standard errors (se). Then the OR_a_ (adjusted OR) for each 10 μg/m^3^ increase in PM_2.5_ exposure can be computed by the following formula:

$$ {\mathrm{OR}}_{\mathrm{a}}=\mathrm{E}\mathrm{X}\mathrm{P}\left(\upbeta \times 10/\mathrm{x}\right) $$

Where *x* (μg/m^3^) is the exposure dose for OR reported in each included study. Similarly, the 95%CI of OR_a_ could also be calculated. We then conducted several meta-analyses of the identified studies to quantitatively estimate the associations of PM_2.5_ exposure during the entire pregnancy and trimester-specific exposure durations with preterm birth risks. Several secondary analyses were also conducted to estimate the pooled-effects of PM_2.5_ exposure during the entire pregnancy on preterm birth in subgroups with different exposure measurement methods, study designs, and study settings. These subgroup analyses aimed to explore the modification effects of these characteristics on the estimates of PM_2.5_ exposure on preterm birth and to further test their impacts on the heterogeneity in the reported associations. Three exposure measurement methods were identified in the included studies: individual-level, semi-individual-level, and regional-level exposure assessment. All these three assessment methods were based on residential level. Individual-level exposure was assessed using complicated dispersion models based on traffic, meteorology, roadway geometry, vehicle emission, air quality monitoring, and land use databases [16, 17]. These models could estimate each subject’s daily PM_2.5_ exposure level with high accuracy. Semi-individual exposure was estimated using the daily PM_2.5_ concentration from the monitoring station nearest to the individual’s residence [7, 8]. Regional-level exposure was calculated using the average PM_2.5_ concentration in a region or a grid with low resolution. This method did not consider the variation in PM_2.5_ concentration within a region, and assumed that all subjects in this region had the same PM_2.5_ exposure concentration. The PM_2.5_ data usually had been obtained from monitoring networks and remote sensing [18]. The study designs of all of the included studies were divided into two categories: retrospective and prospective. In addition, several meta regression analyses were further employed to assess the impacts of study characteristics on the associations between PM_2.5_ exposure and preterm birth risks.

To explore the possible heterogeneity of study results, we hypothesized that effect size may differ according to the methodological quality of the studies. The heterogeneity of the included studies was assessed using the Q statistic and I^2^ statistic. Cochran’s Q statistic was calculated by summing the squared deviations of each study’s estimate from the overall meta-analysis estimate weighted by each study’s contribution. A p-value was obtained by comparing the Q statistic with a chi-square distribution with k-1° of freedom, where k is the number of included studies [19]. If the *p*-value was <0.05, then a random-effects model would be selected, otherwise a fixed-effects model would be selected [20, 21]. The I^2^ statistic [*I*^2^ = (*Q* − *df*)/*Q* × 100] describes the percentage of variation across studies that is due to heterogeneity rather than chance. I^2^ > 50 % demonstrated that there is a statistically significant heterogeneity [19]. We also used funnel plot asymmetry to detect potential publication bias. Egger’s regression was applied to test the funnel plot symmetry, with the inverse of the standard error as the independent variable and the standardized estimate of size effect as the dependent variable [22].

Finally, a series of sensitivity analyses was performed to test the robustness of our results. Because some subgroup analyses included very few studies, we conducted sensitivity analyses only overall and in sub-groups analyses that included more than five studies. For each sensitivity analysis, we individually removed a single study with the largest OR, the smallest OR, the largest standard error, and the smallest standard error from the meta-analyses.

All statistical tests were two-sided, and *P* <0.05 was considered statistically significant. We used R software (version 2.15.2; R Development Core Team 2012, www.R-project.org) to analyze the data.

## Results

### Search results and study characteristics

Twenty-seven potentially eligible studies were identified and assessed for full text. A total of nine studies were excluded for the following reasons: having a different definition of preterm birth (*n* = 1) [23], not providing the dose–response relationship between PM_2.5_ exposure and preterm birth (*n* = 4) [24–27], only assessing the sources of PM_2.5_ (*n* = 1) [28], and duplication of studies whose primary results had already been included in other studies (*n* = 3) [29–31]. Eighteen studies were ultimately included in this meta-analysis, containing a total of more than 3,000 000 subjects with more than 299,000 preterm births [7–10, 16–18, 32–42]. Most studies (12/18) were conducted in the USA [7, 16, 18, 32, 34–40, 42]. There were 12 retrospective and six prospective studies. There were two studies assessing maternal PM_2.5_ exposure at individual, ten at semi-individual, and two at regional levels. The other four studies used two methods to assess PM_2.5_ exposure. The average Newcastle-Ottawa quality score is 8. Detailed information about the included studies is presented in Table 1.

**Table 1:** Characteristics of the studies included in the meta-analysis

Authors	Study setting	Study period	Study design	Exposure assessment level	Data source	No. of participants	No. of cases	Exposure period	Exposure range (mean (IQR) μg/m^3^)	Quality score^a^
Wilhelm et al. [39]	California, USA	1999–2000	Retrospective	Semi-individual level	Monitoring network data	106,483	92,68	TS	21.0 (NA)	8
Huynh et al. [7]	California, USA	1999–2000	Retrospective	Semi-individual level	Monitoring network data	42,692	10,673	WP and TS	18.0 (8.7)	8
Jalaludin et al. [33]	Sydney, Australia	1998–2000	Retrospective	Regional level and semi-individual level	Monitoring network data	123,840	6011	TS	9.0 (4.5)	8
Ritz et al. [37]	California, USA	2003	Prospective	Semi-individual level	Monitoring network data	58,316	5924	TS	20.0 (NA)	7
Brauer et al. [8]	Vancouver, Canada	1999–2002	Prospective	Semi-individual level	Monitoring network data	70,249	3748	WP	5.1 (1.1)	7
Wu et al. [16]	California, USA	1997–2006	Retrospective	Individual level	Monitoring network data	81,186	6712	WP	1.8 (1.4)	9
Gehring et al. [17]	North, west, and center of the Netherlands	1996–1997	Prospective	Individual level	Monitoring network data and land use regression model	3853	165	WP and TS	20.1 (4.6)	7
Rudra et al. [38]	Washington, USA	1996–2006	Retrospective	Semi-individual level	Monitoring network data	3509	369	TS	10.1 (NA)	9
Kloog et al. [34]	Massachusetts, USA	2000–2008	Retrospective	Semi-individual level	Remote sensing data	634,244	61,972	WP	9.6 (5.3)	9
Lee et al. [35]	Pittsburgh, USA	1997–2002	Prospective	Semi-individual level	Monitoring network data	34,705	1940	TS	15.6 (4.0)	7
Chang et al. 2015	Atlanta, USA	1999–2005	Retrospective	Semi-individual level	Monitoring network data	175,891	18,648	WP and TS	17.3 (3.1)	8
Fleischer et al. [10]	22 countries	2004–2008	Retrospective	Regional level	Remote sensing data	192,900	13,379	WP	1.4–98.1 (NA)	7
Nannam et al. 2014 [41]	Northwest England	2004–2008	Retrospective	Semi-individual level and individual level	Monitoring network data	265,613	38,608	WP and TS	22.1 (4.6)	9
Ha et al. [42]	Florida, USA	2004–2005	Retrospective	Regional level and semi-individual level	Monitoring network data	423,719	39,082	WP and TS	9.9 (2.0)	8
Hyder et al. [18]	Connecticut and Massachusetts, USA	2000–2006	Retrospective	Regional level and semi-individual level	Monitoring network data and remote sensing data	647,942	41,868	WP and TS	11.9 (2.4)	8
Gray et al. [32]	North Carolina, USA	2002–2006	Retrospective	Regional level	Monitoring network data	457,642	40,746	WP	13.6 (2.0)	8
Pereira et al. [9]	Connecticut, USA	2000–2006	Prospective	Semi-individual level	Monitoring network data	61,688	-	WP and TS	12.4 (2.3)	9
Pereira et al. [56]	Perth, Australia	1997-2007	Prospective	Semi-individual level	Monitoring network data	31,567	-	WP and TS	8.6 (2.2)	9

NA: Data not available

^a^: Newcastle-Ottawa quality score

-: The number of cases was not available because these studies were longitudinal studies that assessed the effects of PM_2.5_ on preterm birth across successive pregnancies. NA: Data not available

### The pooled effects of PM_2.5_ exposure in different trimesters of pregnancy on preterm birth

**Fig. 2:**
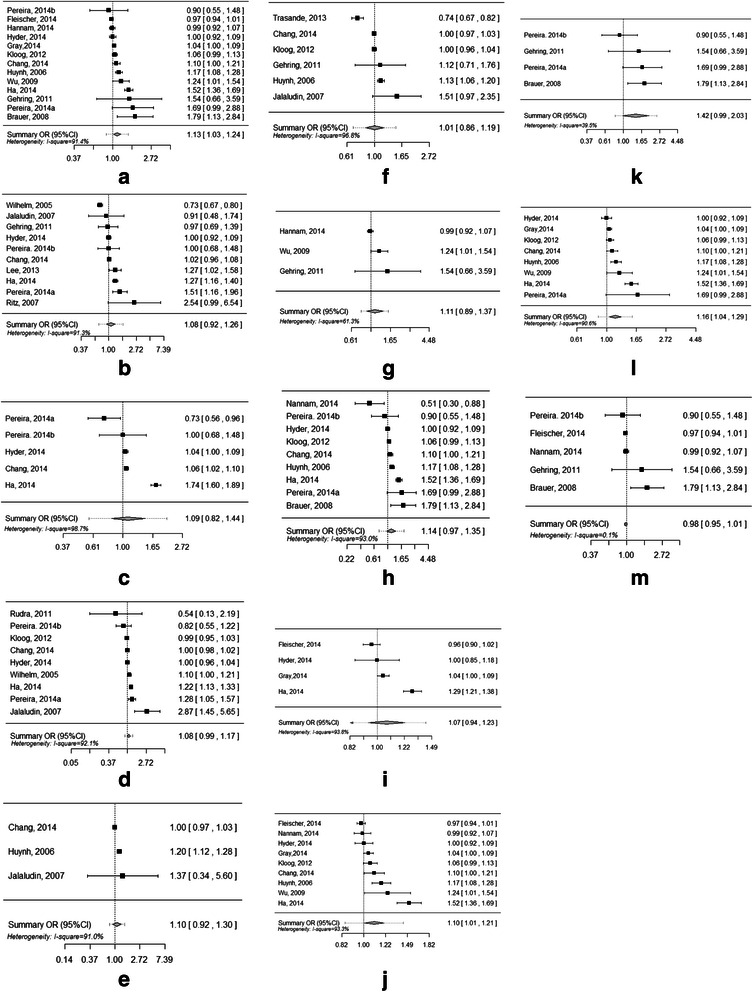
Forest plots for the pooled ORs for the association between PM_2.5_ exposure (per 10 μg/m^3^ increment) during the pregnancy and preterm birth. **a**: In studies that assessed PM_2.5_ exposure during the entire pregnancy. **b**: In studies that assessed PM_2.5_ exposure in the first trimester. **c**: In studies that assessed PM_2.5_ exposure in the second trimester. **d**: In studies that assessed PM_2.5_ exposure in the third trimester. **e**: In studies that assessed PM_2.5_ exposure in the first month of gestation. **f**: In studies that assessed PM_2.5_ exposure within one month before birth. **g**: In studies that assessed PM_2.5_ exposure at individual level. **h**: In studies that assessed PM_2.5_ exposure at semi-individual level. **i**: In studies that assessed PM_2.5_ exposure at regional level. **j**: In retrospective studies. **k**: In prospective studies. **l**: In studies conducted in the USA. **m**: In studies conducted in other countries

We estimated a significant increase of preterm birth risk associated with overall PM_2.5_ exposure (per 10 μg/m^3^ increment) during pregnancy across all 13 included studies (OR = 1.13, 95 % CI = 1.03–1.24) (Table 2 and Fig. 2). The pooled OR values of PM_2.5_ exposure in the first, second and third trimester were 1.08 (95 % CI = 0.92–1.26), 1.09 (95 % CI = 0.82–1.44) and 1.08 (95 % CI = 0.99–1.17), respectively. We did not find any significant effects of PM_2.5_ exposure in either the first month (OR = 1.10, 95 % CI = 0.92–1.30) or the last month of gestation (OR = 1.05, 95 % CI = 0.97–1.13) (Table 2 and Fig. 2).

**Table 2:** Pooled associations between PM_2.5_ exposure (per 10 μg/m^3^ increment) during pregnancy and preterm birth risks in different subgroups

Subgroups	No. of studies	Heterogeneity test		Summary OR (95 % CI)	Hypothesis test		I^2^ (%)	Egger’s test	
		Q	P		Z	P		t	P
Exposure during the entire pregnancy	13	80.51	<0.001	1.13* (1.03–1.24)	2.59	0.010	91.4	2.20	0.051
Specific trimester									
First trimester exposure	10	89.14	<0.001	1.08 (0.92–1.26)	0.96	0.334	91.3	0.68	0.517
Second trimester exposure	5	138.69	<0.001	1.09 (0.82–1.44)	0.60	0.548	98.7	0.311	0.776
Third trimester exposure	9	44.83	<0.001	1.08** (0.99–1.17)	1.70	0.089	92.1	1.58	0.157
First month of gestation	3	22.03	<0.001	1.10 (0.92–1.30)	1.03	0.301	91.0	0.58	0.666
Within one month before birth	6	51.49	<0.001	1.01 (0.86–1.19)	0.09	0.926	96.8	0.03	0.980
Exposure assessment method^a^									
Individual exposure	3	4.94	0.085	1.11 (0.89–1.37)	0.93	0.352	61.3	1.74	0.332
Semi-individual exposure	9	55.86	<0.001	1.14 (0.97–1.35)	1.56	0.119	93.0	0.35	0.737
Regional level	4	46.19	<0.001	1.07 (0.94–1.23)	1.00	0.319	93.8	0.11	0.921
Study design^a^									
Retrospective studies	9	70.98	<0.001	1.10* (1.01–1.21)	2.12	0.034	93.3	2.31	0.055
Prospective studies	4	4.64	0.201	1.42* (1.08–1.85)	2.52	0.012	39.5	0.10	0.927
Study setting^a^									
USA	8	50.49	<0.001	1.16* (1.05–1.29)	2.73	0.006	90.6	1.80	0.121
Others	5	7.90	0.095	0.98 (0.95–1.01)	1.11	0.268	0.1	1.62	0.205

^a^: All of these subgroup analyses were conducted for the studies that assessed the association between PM_2.5_ exposure during the entire pregnancy and preterm birth. All of these estimates were ORs for each 10 μg/m^3^ increment of PM_2.5_ exposure during the entire pregnancy

*: *p* < 0.05

**: 0.05 < *p* < 0.10

### Subgroup analyses on the effects of exposure assessment methods, study designs and study settings on the associations between PM_2.5_ exposure during the entire pregnancy and preterm birth

We found numerically similar pooled associations between preterm birth risk and PM_2.5_ exposure in studies using different exposure assessment methods. The pooled ORs in the studies that assessed PM_2.5_ exposure at individual, semi-individual and regional levels were 1.11 (95 % CI = 0.89–1.37), 1.14 (95 % CI = 0.97–1.35) and 1.07 (95 % CI = 0.94–1.23), respectively (Table 2 and Fig. 2).

We observed significant pooled estimates of PM_2.5_ on preterm in studies that used a retrospective (OR = 1.10, 95 % CI = 1.01–1.21) or prospective study design (OR = 1.42, 95 % CI = 1.08–1.85). Furthermore, the latter meta-estimate of PM_2.5_ was larger than the former (Table 2 and Fig. 2).

The pooled estimate of the association between PM_2.5_ exposure and preterm birth was statistically significant for studies that were conducted in the USA (OR = 1.16, 95 % CI = 1.05–1.29), but the pooled estimate was not significant for studies that were conducted in other countries (OR = 0.98, 95 % CI = 0.95–1.01) (Table 2 and Fig. 2).

In addition, several meta regression analyses were employed to further evaluate the impacts of study characteristics on the associations between PM_2.5_ exposure and preterm birth risks (Additional file 1: Figure S1). We observed similar results with the subgroup analyses. For instance, the combined estimate of PM_2.5_ exposure during the entire pregnancy were higher in prospective studies than in retrospective studies (β = 0.25, *P* = 0.120).

### Sensitivity analyses on the associations between PM_2.5_ exposure and preterm birth

**Fig. 3:**
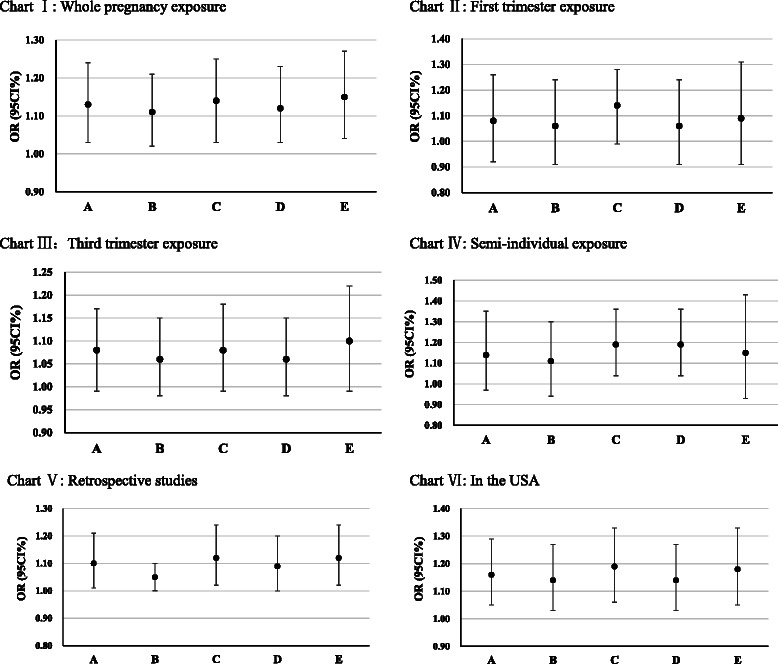
Sensitivity analysis for the pooled effects of PM_2.5_ exposure (per 10 μg/m^3^ increment) on preterm birth risk in different subgroups. *a*: All studies were included; *b*: The study with the largest effect size was excluded; *c*: The study with the smallest effect size was excluded; *d*: The study with the largest standard error was excluded; *e*: The study with the smallest standard error was excluded. Chart I: In studies that assessed the association between PM_2.5_ exposure during the entire pregnancy and preterm birth. Chart II: In studies that assessed the association between PM_2.5_ exposure in the first trimester and preterm birth. Chart III: In studies that assessed the association between PM_2.5_ exposure in the third trimester and preterm birth. Chart IV: In studies that assessed the PM_2.5_ exposure at the semi-individual level. Chart V: In retrospective studies. Chart VI: In studies that were conducted in the USA

In the meta-analysis that included studies assessing PM_2.5_ exposure at the semi-individual level, the PM_2.5_ meta-estimate became significant after excluding a single study with the smallest effect size. Beyond that, we did not find any significant change in the PM_2.5_ meta-estimates in other meta-analyses after excluding a single study with the largest effect size, the smallest effect size, the largest standard error, or the smallest standard error (Fig. 3).

### Heterogeneity and publication bias

**Fig. 4:**
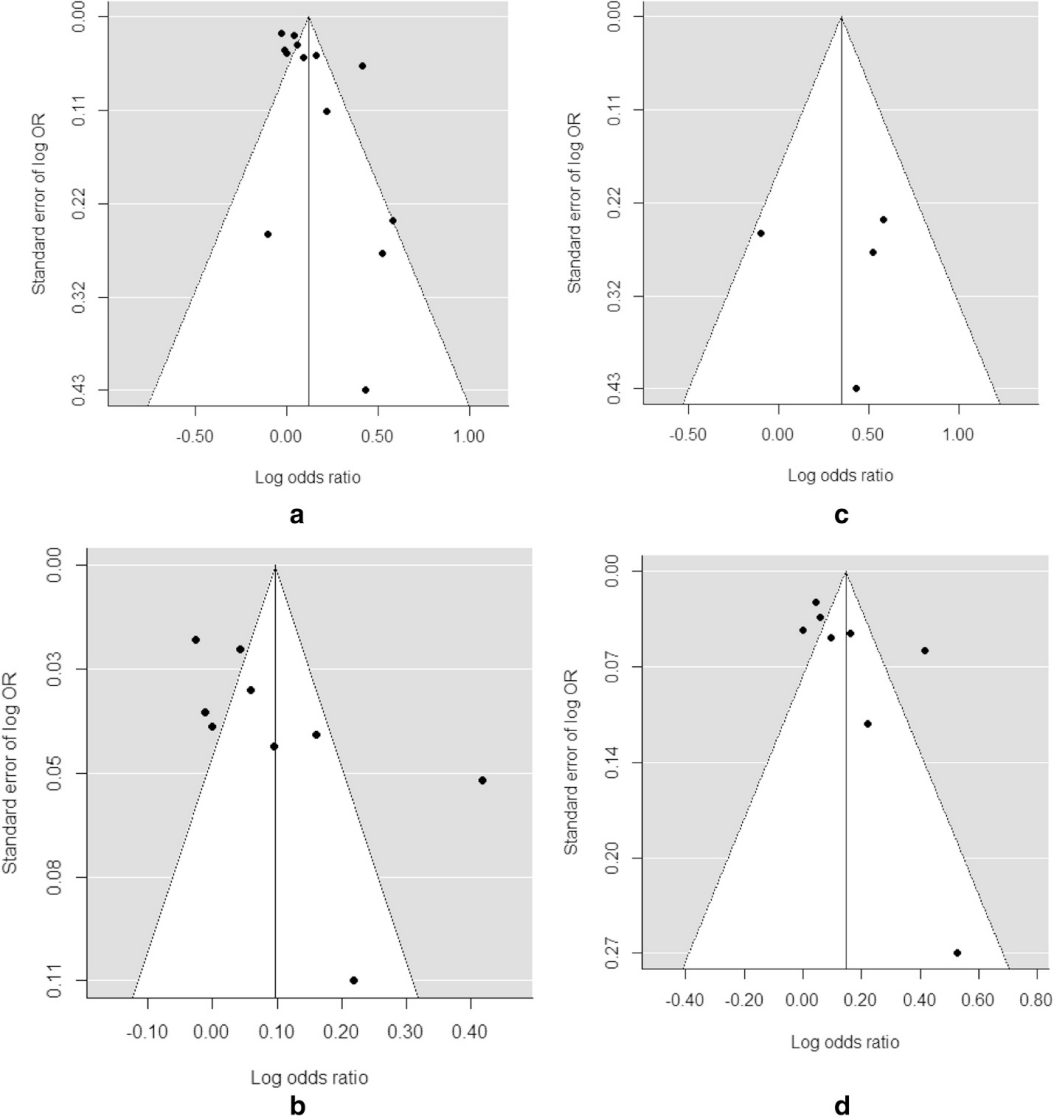
Funnel plots for the meta-analyses assessing the associations between PM_2.5_ exposure (per 10 μg/m^3^ increment) during pregnancy and preterm birth. **a**: In studies that assessed PM_2.5_ exposure during the entire pregnancy. **b**: In retrospective studies. **c**: In prospective studies. **d**: In studies conducted in the USA

We observed significant heterogeneities in most of the meta-analyses. However, in some subgroup analyses, such as the subgroup of prospective studies, there were no significant heterogeneities between studies. These findings indicate that the three characteristics that we took into account in this study were important sources of heterogeneities between studies. We did not find any statistically significant publication bias in any of the meta-analyses (Table 2 and Fig. 4).

## Discussion

In this meta-analysis, we quantitatively assessed the association between maternal PM_2.5_ exposure during pregnancy and preterm birth risk. We observed a clearly significant association between PM_2.5_ exposure during pregnancy and preterm birth risk, which is consistent with the results of previous meta-analyses [11, 13]. Sapkota et al. estimated a pooled OR of 1.15 (95 % CI = 1.14–1.16) for preterm birth per 10 μg/m^3^ increment in PM_2.5_ exposure during the entire pregnancy [11]. Zhu et al. reported that a 10 μg/m^3^ increase in PM_2.5_ exposure over the entire pregnancy was positively associated with a 10 % (95 CI % = 3.0–18 %) increase in preterm birth risk [11] Stieb et al.’s meta-analysis also found a positive but non-significant association between PM_2.5_ exposure and preterm birth (OR = 1.05, 95 CI % = 0.98–1.13). The lack of statistical significance may be due to the small quantity of included studies (*n* = 4) [12]. These results further demonstrate the adverse effect of PM_2.5_ exposure during pregnancy on preterm birth. Air pollution is ubiquitous, and all populations are exposed to it at some level. Immature fetuses are more susceptible to air pollution [43]. Therefore, these results are important for policy makers and public health practitioners worldwide.

Although the mechanisms of PM_2.5_ leading to preterm birth are not well understood, inhaled PM_2.5_ can penetrate the gas exchange region of the lungs and enter the bloodstream. Toxic chemicals such as carcinogenic polycyclic aromatic hydrocarbons and harmful metals could cause systemic oxidative stress, oxidative stress-induced DNA damage, pulmonary and placental inflammation, blood coagulation, endothelial dis-function, and hemodynamic changes [44]. These responses could interfere with the transplacental oxygen and nutrient transport from mothers to fetuses, and has the potential to negatively impact fetal growth and development, particularly during critical periods of organogenesis [43, 45, 46]. In addition, The early activation of cytokines favoring inflammation may play an important role in the PM_2.5_-preterm link, because inflammatory mediators such as interleukin 1-β (IL-1β) and tumor necrosis factor-α (TNF-α) can trigger the premature onset of labor [46].

The question of which gestational windows are more susceptible to air pollution has been explored in several previous studies. Although some studies supported early pregnancy (the first month or first trimester) [7, 35, 37], other studies supported later pregnancy (the third trimester, the last month, or the last week) [33, 39] as the window of susceptibility. A meta-analysis also observed a pronounced association between PM_2.5_ exposure during the third trimester and preterm risk [11]. It was debated that PM_2.5_ exposure during the later pregnancy might induce early activation of cytokines favoring inflammation, and trigger the premature onset of labor [46]. By contrast, the implantation of the fetus and the formation of the placenta occur during the first trimester, and higher PM_2.5_ exposure during this time period might cause genetic mutations, and hence increase the risks of fetal malformation, miscarriage and even death [47]. These serious harmful effects might attenuate the association between PM_2.5_ exposure in early pregnancy and preterm outcome. However, in this study we observed nearly identical pooled estimates of PM_2.5_ exposure during the first, second and third trimester, which indicates that more studies are needed in the future to explore which gestational windows are more susceptible to air pollution.

Exposure assessment is an important issue in studies estimating the effects of ambient air pollution on health. In this meta-analysis, we selected studies that assessed PM_2.5_ exposure at the individual, semi-individual or regional level. Using monitoring data for nearby areas or regional average PM_2.5_ concentrations measured at monitoring stations may provide a misrepresentation of exposure because this method does not take into account the spatial misalignment between an individual’s residence and monitoring sites, and ignores the fact that individuals have different activity models (indoor and outdoor activity time) and could have changed their residential address during pregnancy [8, 48, 49]. Some recent studies used complicated dispersion models to quantitatively assess individual PM_2.5_ exposure [16, 17]. These models included data on several variables including traffic, meteorology, roadway geometry, vehicle emission, air quality, and land use. However, the accessibility of these datasets usually limits the wide employment of these dispersion models, particularly in some developing countries where the information on land use, traffic and vehicle emission is limited. In recent years, some studies used personal monitors to assess maternal exposure to air pollutants in different trimesters [50, 51]. These methods could theoretically reduce the bias in exposure assessment. In this study, although we observed stronger pooled associations between PM_2.5_ exposure and preterm birth in studies that assessed PM_2.5_ exposure at the individual and semi-individual levels than for studies that used regional-level methods, the lack of significant associations indicate that more studies are needed in the future, especially studies assessing PM_2.5_ exposure at the individual level. For example, we only included three studies that used the individual-level assessment method, and their pooled estimate was dominated by a single study.

It is well known that the toxicity and health impacts of PM_2.5_ may vary by geographic area [52]. Therefore, it is reasonable to conduct subgroup meta-analyses to test the variation in PM_2.5_ estimates between regions. In this study, because most of the included studies were conducted in the USA, we divided all studies into two groups (USA and other countries). However, we found a significant meta-estimate of PM_2.5_ exposure only for the US studies. This discrepancy may be partially related to the small number (*n* = 5) of studies in the second group, which indicates that more studies in countries other than the USA are needed, especially in middle or low income countries with higher levels of air pollution. For example, only one study has been found that assessed the association between PM_2.5_ exposure and preterm birth in China and India. These two countries have the most severe PM_2.5_ pollution [53], and the largest number of preterm births worldwide [1]. Studies in these countries could provide important information for policy makers and public health practitioners to reduce the health impacts of air pollution.

Although this meta-analysis estimated the pooled effects of PM_2.5_ concentrations on preterm birth risks, the limited number of studies restricted us from further exploring the effects of the chemical components of PM_2.5_ on preterm birth. PM_2.5_ is a mixture of multiple inorganic and organic components, and its health effects can vary based on components and origins [54, 55]. Only two of the included studies assessed the association between the components and sources of PM_2.5_ and preterm birth. Pereira et al. observed that preterm birth in Connecticut, USA was associated with increased exposure to dust, motor vehicle emissions, oil combustion and regional sulfur PM_2.5_ sources during the entire pregnancy [56]. Darrow et al.’s study in Atlanta, USA found that preterm birth was significantly associated with sulfates and water-soluble metals in PM_2.5_, but not associated with other components [30]. These results demonstrate that studies on the association between PM_2.5_ components and sources and preterm birth are still limited, and more studies are needed in the future.

Another factor affecting the heterogeneity between studies is the way that the studies controlled for confounders [57]. All of the studies included in this meta-analysis provided adjusted estimates of PM_2.5_ exposure. Some common confounders, such as maternal age, race/ethnicity, income, education, smoking status during pregnancy, infant sex, parity, and birth season, were adjusted for in most studies. However, almost all of the maternal and infant information was from public records, such as birth certificates, which limited the ability to control for other important confounders, such as maternal stress, activity level, nutrition, indoor pollution, and factors that varied spatially [36, 50, 51]. Therefore, improving the data quality of public records is one way to improve related studies. Future longitudinal studies that collect more detailed information at the individual level would be beneficial.

With reference to the limitations of this meta-analysis, we found high heterogeneity between included studies. Therefore, we used a random-effects model to quantitatively combine the individual estimate in studies with high heterogeneities. We also employed subgroup analyses and meta-regression analyses to explore the sources of heterogeneity. The results showed that although exposure assessment methods, study designs and study settings partially explained the heterogeneity, significant heterogeneities were still found in most subgroup analyses. These findings indicate that the heterogeneity across the included studies may also have been affected by other factors that we did not consider in this study, such as socioeconomic status and chemical constituents of PM_2.5_, due to the limited quantity of related studies. Therefore, further studies are needed to explore the sources of heterogeneity in the future.

## Conclusions

In summary, this meta-analysis observed a clear association between PM_2.5_ exposure during pregnancy and preterm birth risk. However, a significant heterogeneity was found between included studies. The exposure assessment method, study design and study setting might be important sources of the heterogeneity, and should be taken into account in future meta-analyses. This study extends our understanding of the effects of maternal PM_2.5_ exposure on preterm birth, and highlights that it is crucial to reduce ambient PM_2.5_ pollution and reduce maternal PM_2.5_ exposure during pregnancy to improve birth outcomes.

## Additional file

Additional file 1: Figure S1. Meta regression analyses on the effects of study characteristics on the associations between PM_2.5_ exposure and preterm birth risks. Chart A: The effects of PM_2.5_ exposure during different trimester (the first, second and third trimester) of pregnancy on the pooled estimate of PM_2.5_. Chart B: The effects of PM_2.5_ exposure assessed by different methods (regional, semi-individual and individual level) on the pooled estimate of PM_2.5_. Chart C: The effects of study designs (retrospective and prospective studies) on the pooled estimate of PM_2.5_. Chart D: The effects of study settings (the USA and other countries) on the pooled estimate of PM_2.5_ (DOCX 56 kb)

## Abbreviations

PM_2.5_: Fine particulate matter with aerodynamic diameter <2.5 μm
OR: Odds ratio
CI: Confidence interval
USA: The United States of America
PTB: Preterm birth
PTD: Preterm delivery
NA: Data not available
