# Supplementary figures and images for: The association between fine particulate matter exposure during pregnancy and preterm birth: a meta-analysis
Source: BMC Pregnancy Childbirth. 2015 Nov 18;15:300. doi: 10.1186/s12884-015-0738-2 (PMC4650291; doi:10.1186/s12884-015-0738-2)

| 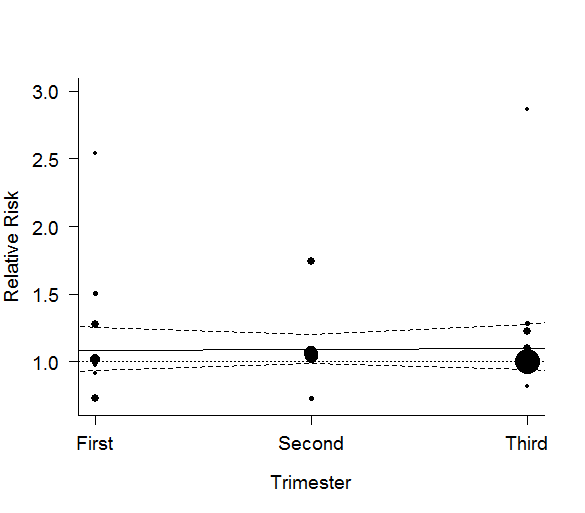  A | 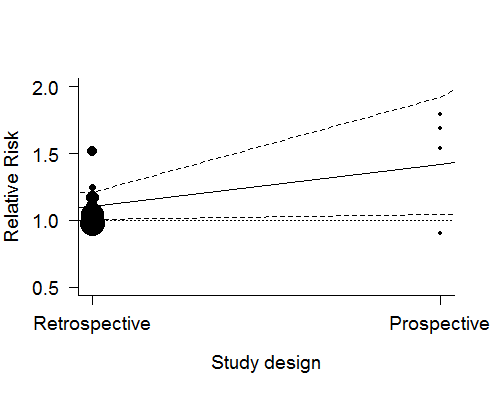  C |
| --- | --- |
| 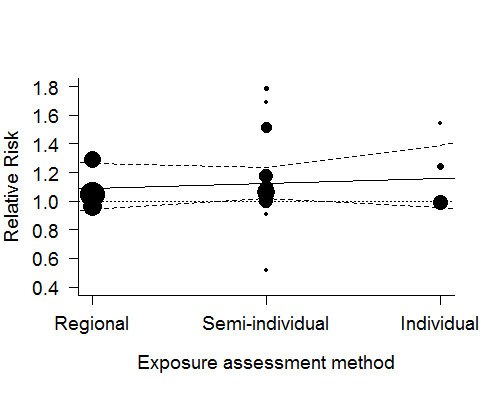  B | 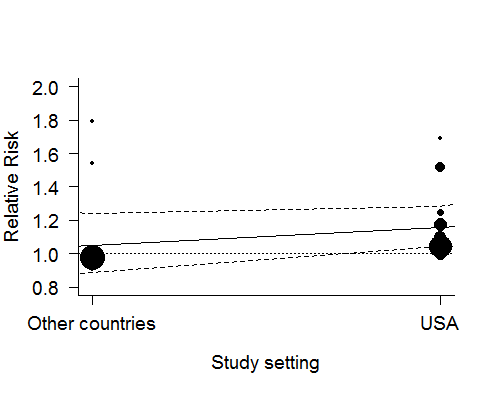  D |

Supplement: Additional file 1: Figure S1. — Meta regression analyses on the effects of study characteristics on the associations between PM2.5 exposure and preterm birth risks. Chart A: The effects of PM2.5 exposure during different trimester (the first, second and third trimester) of pregnancy on the pooled estimate of PM2.5. Chart B: The effects of PM2.5 exposure assessed by different methods (regional, semi-individual and individual level) on the pooled estimate of PM2.5. Chart C: The effects of study designs (retrospective and prospective studies) on the pooled estimate of PM2.5. Chart D: The effects of study settings (the USA and other countries) on the pooled estimate of PM2.5 (DOCX 56 kb) [file 12884_2015_738_MOESM1_ESM.docx]
